# Waste Orange Peels as a Feed Additive for the Enhancement of the Nutritional Value of *Tenebrio molitor*

**DOI:** 10.3390/foods12040783

**Published:** 2023-02-12

**Authors:** Konstantina Kotsou, Theodoros Chatzimitakos, Vassilis Athanasiadis, Eleni Bozinou, Christina Adamaki-Sotiraki, Christos I. Rumbos, Christos G. Athanassiou, Stavros I. Lalas

**Affiliations:** 1 Department of Food Science and Nutrition, University of Thessaly, Terma N. Temponera Str., 43100 Karditsa, Greece; 2 Laboratory of Entomology and Agricultural Zoology, Department of Agriculture, Crop Production and Rural Environment, School of Agricultural Sciences, University of Thessaly, Phytokou Str., 38446 Volos, Greece

**Keywords:** edible insects, *Tenebrio molitor*, orange albedo, proximate composition, carotenoids, vitamin A, vitamin C, proteins, fat, fatty acids

## Abstract

Lately, additional attention is being placed on edible insects, since they constitute an excellent, cost-efficient source of proteins with a low ecological footprint. *Tenebrio molitor* was the first insect that was considered edible by EFSA in 2021. This species can replace conventional protein sources and thus, it has the potential to be used in many different food products. In the present study, a food by-product that is commonly produced (i.e., albedo orange peel waste) was used as a feed additive for *T. molitor* larvae, in an effort to further improve the circular economy and enhance the nutritional value of the insects. To this end, bran, which is commonly used as feed for *T. molitor* larvae, was fortified with the albedo orange peel waste (up to 25% *w*/*w*). Larval performance, in terms of larval survival and growth, as well as the larval nutritional value, i.e., the content of protein, fat, carbohydrates, ash, carotenoids, vitamins A and C, and polyphenols, was evaluated. Based on the results, the increase in the percentage of orange peel albedos in *T. molitor* feed resulted in a subsequent increase in the content of larvae in carotenoids and vitamin A up to 198%, in vitamin C up to 46%, and an increase in the protein and ash content up to 32% and 26.5%, respectively. Therefore, the use of albedo orange peel waste for feeding of *T. molitor* larvae is highly recommended, since it results in larvae with enhanced nutritional value and at the same time, the utilization of this feeding substrate further lowers the cost of insect farming.

## 1. Introduction

As the world population increases (it is estimated to reach ~10 billion by 2050) the demand for food will increase. This will be accompanied by higher demand for protein-rich foods, in order to sustain a healthy life. Currently, the main source of protein is from animals. Around 350 million tons of meat are produced annually and the estimation is an increase of ~60 million tons by 2050 [1]. To sustain this livestock production, nearly 75% of the total arable land is used [2]. However, intensive livestock farming and its industrialization have a negative environmental impact, since the livestock sector is responsible for 14.5% of total greenhouse gas emissions [3]. Therefore, doubts are rising about the sustainability of livestock farming and the cover of the increasing needs. Hence, alternative sources of protein are being exploited, to address the issue and avoid malnutrition. To this end, insects have emerged as a promising option [4]. Insects have attracted the interest of researchers, primarily due to their high nutritional value. In addition, since their growth requires less space, water, and feed, as well as the fact that insects have a fast life cycle, they exhibit major economic and ecological advantages, rendering them not only a suitable alternative to animal-derived protein but also advantageous, when compared to chicken, pork, and beef meat [5].

To this end, many insects have been studied to examine whether they are edible. Among the ~2000 edible insect species, the yellow mealworm, *Tenebrio molitor* L. (Coleoptera: Tenebrionidae), is one of the most thoroughly investigated insect species and it is an excellent, cost-efficient source of proteins with a low ecological footprint [6]. This was the reason why it was considered edible by EFSA in 2021 and became the first, officially accepted, edible insect in Europe (European Commission 2021/882) [7], even though it was already used for animal feeds. *Tenebrio molitor* larvae can be eaten whole or processed to produce flour, extracts, and oils, which can be used to reinforce biscuits, snacks, pasta, etc. [6]. On average, *T. molitor* larvae contain about 20–50% of dry weight proteins, 20–45% fat, 5–20% carbohydrates, and 1–4% ash [8]. However, these values can vary a lot, depending on the developmental stage of the larvae, the sex, the growth environment, and mainly, their diet [9,10,11]. The optimum conditions for the survival and development of all life stages of *T. molitor* are 25 °C and 75% relative humidity, whereas its diet consists mainly of flour, bran, cereals, and bread [12,13,14].

In an effort to produce larvae with high nutritional value, various feeds have been exploited. For instance, Kröncke et al. [10] examined 25 substrates used as feed for *T. molitor* larvae and found that substrates such as rice protein flour, lupine flour, and potato flakes had a positive impact on the biomass growth of the larvae. Raspberry seed cake, dried sugar beet pulp, and coconut flour should be added in small amounts, while others (e.g., mustard flour and psyllium husks) must be avoided, since they can contain compounds that exhibit insecticide activity. Therefore, the selection of the proper substrate is of paramount importance. This is also strengthened by the fact that the mass production of the larvae is expensive, and as such, feeds of low cost are needed to make the production more profitable. To this end, a wide spectrum of organic side streams has been evaluated so far as an alternative option [15]. Indicatively, about 90 million tons of food waste are produced annually in the European Union [16]. Therefore, they could consist of an inexpensive, easily found source of nutrients for insects. Since these side-streams are unsuitable for human consumption, their use for insect rearing will not compete with the human diet, as is the case for other animals. Additionally, their disposal is a major environmental and economic issue. Therefore, valorizing organics side-streams, such as former foodstuff products [17], by-product meals from vegetable oil production [18], and by-products of the cereal and legume seed cleaning process [19], leads toward the development of a circular economy system that exhibits multiple benefits. 

Taking into account that organic side-streams can be an excellent source of nutrients and bioactive compounds, they could be upcycled to promote larval growth and simultaneously increase their overall nutritional value to be used for human diets. In most studies, special emphasis is placed on the increase in protein content, while the increase in other nutrients, such as vitamins, is often overlooked. In the present study, a food by-product that is commonly produced (i.e., waste orange peel) was valorized as feed for *T. molitor* larvae, in an effort to enhance their nutritional value and propose an alternative way of valorizing the orange peels. The albedo of the peels was added to the bran feed and the larvae were fed to examine whether their nutritional value could be increased. Additionally, the contents of vitamins A and C were examined, since orange peel albedos are an excellent source of both vitamins [19]. Albedos were chosen, since they are less commonly employed for other uses, compared to flavedos, which are used for culinary, zesting, and pastry purposes.

## 2. Materials and Methods

### 2.1. Chemicals

All solvents used were of HPLC grade and were purchased from Carlo Erba (Val deReuil, France). Gallic acid, sodium anhydrous carbonate, 2,2-diphenyl-1-picryl-hydrazyl (DPPH), 2,4,6-tri-2-pyridinyl-1,3,5-triazine (TPTZ), and Folin–Ciocalteu reagent were received from Penta (Prague, Czech Republic). Hydrochloric acid, Bradford reagent, iron (III) chloride, ascorbic acid, β-carotene, and trichloroacetic acid were purchased from Sigma-Aldrich (Steinheim, Germany).

### 2.2. Insects

Stock colonies of *T. molitor* were reared in plastic insect breeding trays (60 × 40 × 14.5 cm) (Beekenkamp Verpakkingen BV, Maasdijk, The Netherlands). The boxes were lacking the top cover to allow air circulation and box aeration. The stock colonies were maintained in a pilot-scale insect-rearing unit at the Laboratory of Entomology and Agricultural Zoology at the University of Thessaly, Greece under constant conditions (i.e., 27 ± 0.5 °C, 60 ± 5% relative humidity and continuous darkness).

To obtain newly emerged larvae, 250 g of adults were allowed to oviposit for 7 d in crates (60 × 40 × 14.5 cm) along with 2 Kg of wheat flour. Adults were placed on the top of a mesh to avoid cannibalism of eggs. Agar was provided to adults ad libitum as a moisture source. After the 7-day interval, adults were removed and the newly emerged larvae were left to feed on flour for 14 d before the initiation of the experiments. Larvae were separated from the flour by sieving. To determine the available amount of larvae, 6 subsamples (with at least 100 larvae) were taken and the initial individual larval weight as well as the number of larvae per weight, were estimated.

### 2.3. Feeding Trial 

The insects that served as control were fed with wheat bran (OA0) (purchased from a local market, Volos, Greece) with a particle size smaller than 2 mm. For the different dietary treatments, orange albedo flour was added to the wheat bran at three rates (i.e., 10 (OA10), 17.5 (OA17.5), and 25% (OA25) *w*/*w*). In order to prepare the orange albedo flour, oranges (bought from a local market, Karditsa, Greece) were rinsed with water and dried with a paper towel. Then, the peel was removed, and the albedo was separated from the flavedo manually. The albedos were cut into smaller pieces (~2 × 2 cm) and placed in a freeze-dryer (Biobase BK-FD10P freeze-dryer (Jinan, China)) for 24 h. The dried albedos were crushed to a fine powder and kept frozen (−20 °C) until further use.

Plastic insect breeding trays (24 × 29.5 × 10 cm) were used as experimental units for the feeding trials. Each tray was filled with 500 g of feed (either wheat bran or bran fortified with albedo orange peel waste) and 2500 14 day-old larvae. For each dietary treatment, four tray replicates were prepared. During larval development, agar was provided to larvae three times per week as a moisture source, increasing the amount of agar provided over time, in order to ensure agar availability for larvae. Larvae were left to feed undisturbed. At the end of each week (from 3rd week onwards) the content of each tray was homogenized by gently pouring the content of the tray into an empty container and repeating this procedure five times. Two random subsamples were subsequently taken from each tray. Afterwards, for each subsample the number of larvae was determined, as well as the total larval weight using an analytical balance (Equinox EAB125i, Adam Equipment Inc., Fox Hollow Road, Oxford, England). Care was taken, so that each subsample contained at least 100 larvae. To determine the average individual larval weight, the total larval weight was divided by the number of the larvae in each subsample. At the end of the 6th week, the larvae were harvested in order to carry out the analyses described in Section 2.4. After harvesting, larvae were fasted for 24 h, separated into batches, weighed, and euthanized by freezing (−20 °C). The larvae harvested at the end of the trial were then placed in a freeze dryer for 24 h. The dried larvae were then crushed to a fine powder and stored in amber-glass vials at −20 °C until further analysis.

### 2.4. Larval Composition Analysis

#### 2.4.1. Water Content Calculation

The water contained in the larvae was determined by subtracting the weight after the freeze-dryer process from the weight of the same batch, before freeze-drying.

#### 2.4.2. Crude Protein Content 

The protein content of the samples was determined using the Bradford assay [20]. To extract the proteins from the samples, 10 g of distilled water was added to 1 g of the sample. The pH of the distilled water was previously adjusted to 12, using NaOH solution (1 M). Extraction was carried out by stirring the mixture at 500 rpm for 1 h at room temperature. Next, the mixture was centrifuged for 5 min at 4500 rpm (at room temperature). The supernatant was collected and 10 g of distilled water (pH adjusted to 12) was added to the solid residue. The extraction procedure was repeated two more times (three times in total) and the supernatants were combined. For the determination of the protein content, 100 μL of the combined supernatants was transferred to an Eppendorf tube and 900 μL of Bradford reagent was added. After vortexing for 30 sec, the samples were left to incubate for 10 min at room temperature in the absence of light. Then, the absorbance of the samples was measured at 595 nm using a spectrophotometer (Shimadzu UV-1700 PharmaSpec Spectrophotometer, Kyoto, Japan). A standard calibration curve was prepared using bovine serum albumin.

#### 2.4.3. Carbohydrates

The carbohydrates were determined using the phenol/sulfuric acid assay [21]. To extract carbohydrates, 10 g of distilled water was added to 1 g of the sample. Extraction was carried out by stirring the mixture at 500 rpm for 1 h at 50 °C. Next, the mixture was centrifuged for 5 min at 4500 rpm (at room temperature) and the supernatant was collected. The supernatant (0.22 mL) was transferred to a plastic tube and immediately concentrated in sulfuric acid (0.65 mL) and phenol solution (0.13 mL) (5% *w*/*v* in distilled water). The mixture was placed in a water bath at 90 °C for 5 min and then left to cool at room temperature for 5 min. Finally, the absorbance of the solution was measured at 495 nm using a spectrophotometer. A calibration curve was prepared using D(+)-glucose as a standard.

#### 2.4.4. Ash

The crude ash content of *T. molitor* larvae was found using a gravimetric approach. In a porcelain crucible, an accurately weighted portion of ~5 g dried insect powder was added and placed in an oven. The temperature was increased at a rate of 5 °C/min up to 550 °C. Samples were left in the oven for about 5 h until no black residue could be observed. Then, the samples were placed in a desiccator and left to cool to room temperature. The weight of the crucibles was determined, and, after calculations, the ash content was determined.

#### 2.4.5. Total Fat, Fatty Acids, and Calculated Oxidizability Value (COX)

To determine the fat content of the larvae, a defatting process was carried out. In a screw-capped, amber-glass vial, 1 g of sample was added along with 10 mL of hexane. The mixture was stirred at 600 rpm for 60 min at 40 °C. The mixture was centrifuged at 4500 rpm for 5 min. The supernatant was transferred to a pre-weighted flask and the defatting process was repeated two more times to the solid residue. Each time, the supernatants were transferred to the flask. The solvent was removed using a rotary evaporator and the weight of the extracted oil was calculated.

To determine the fatty acids contained in the extracted oil, fatty acid methyl esters (FAMEs) were prepared according to Commission Regulation (EC) No 796/2002 (Annex XB) [22]. The analysis of methyl esters with GC-FID was carried out according to a modified method described by Lalas et al. [23]. An Agilent Technologies (Santa Clara, CA, USA) Gas Chromatograph model 7890A, equipped with a capillary column Omegawax (30 m × 320 μm × 0.25 μm) (Supelco, Bellefonte, PA, USA) was used. Helium was the carrier gas at a flow rate of 1.4 mL/min. The column temperature program was as follows: initially isotherm for 5 min at 70 °C, an initial programmed rate of 20 °C/min up to 160 °C, then a second rate of 4 °C/min up to 200 °C, and a final rate of 5 °C/min up to 240 °C. The injector and flame ionization detector (FID) temperatures were maintained at 240 and 250 °C, respectively. The flow rate for hydrogen was 50 mL/min, for air 450 mL/min, and the makeup flow of helium was 50 mL/min. Samples of 1 μL were injected into the split mode (1:100). The individual peaks were identified by comparison of reference standards of FAME Mix C8–C24 (Sigma-Aldrich, St. Louis, MO, USA). The percentage composition of the samples was computed from the peak areas using the normalization method (without correction factors). The component percentages were calculated as mean values from triplicate GC-FID analysis [22,24,25]. 

The calculated oxidizability value (COX) was also measured using the method described by Fatemi and Hammond [25], as shown below:

(1) $$\mathrm{COX}=\frac{1\;\left(18:1,\;\%\right)+10.3\;\left(18:2,\;\%\right)+21.6\;(18:3,\;\%)}{100}$$

where 18:1 represents the % percentage of oleic acid, 18:2 of ω-6, linoleic acid, and C18:3 the percentage of ω-3, linolenic acid.

#### 2.4.6. β-Carotene–Vitamin A

β-Carotene and vitamin A content were estimated using a previously reported method [26]. For the extraction step, 1 g of each sample was extracted using 10 mL of ethanol for 30 min at 300 rpm at room temperature, and then, they were placed in an ice bath for 5 min with occasional shaking. Then, the mixture was centrifuged for 5 min at 4500 rpm. The absorbance of the extract was read at 450 nm and the β-carotene content was calculated from a standard β-carotene curve using the interpolation method. Vitamin A was determined using a conversion factor proposed by the United States Department of Agriculture (USDA). Vitamin A: 1 International Unit (IU) = 0.60 μg β-carotene.

#### 2.4.7. Vitamin C

The ascorbic acid content was determined using a modified chromatometric assay [27]. A quantity of 5 g of ground insect was weighed and placed in a beaker, followed by the successive addition of 27 mL distilled water: methanol mixture (60:40, *v*/*v*) and 3 mL of 10% *w*/*v* trichloroacetic acid solution. After vortexing for 1 min, 20 mL of hexane was added. Then, the mixture was further stirred for 30 min at room temperature, followed by centrifugation at 4500 rpm for 5 min. The lower aqueous phase was transferred to a centrifugal tube and centrifuged for 10 min at 10,000 rpm. In an Eppendorf tube, 1 mL of the aqueous layer was transferred, followed by the addition of 0.5 mL Folin–Ciocalteu reagent (10% *v*/*v*), and the mixture was incubated for 10 min at room temperature. Finally, the absorbance was measured at 760 nm. Quantification was carried out by preparing a calibration curve using ascorbic acid.

#### 2.4.8. Total Polyphenol Content (TPC) Determination

For the determination of the total polyphenol content (TPC) of the extracts, a previously reported method was employed [28]. In brief, 100 μL of *T. molitor* extracts (prepared as described in Section 2.4.7) were mixed with an equal portion of the Folin–Ciocalteu reagent. After 2 min, 800 μL of Na_2_CO_3_ solution (5% *w*/*v*) was added and the solution was incubated at 40 °C for 20 min in the absence of light. Finally, the absorbance was measured at 740 nm. Quantification was carried out using a standard calibration curve with gallic acid. The TPC of the solution was expressed as mg of gallic acid equivalents (GAE) per L. Next, the extraction yield of total polyphenols (*Y*_TP_) (expressed as mg GAE per g dry weight) was calculated using the following equation:

(2) $$Y_{\mathrm{TP}}\;(\mathrm{mg}\;\mathrm{GAE}/g\;\mathrm{dw})=\frac{C_{\mathrm{TP}}\;\times \;V}{w}$$

where *V* is the volume of the extraction medium (in L) and *w* is the dry weight of the sample (in g).

#### 2.4.9. Ferric-Reducing Antioxidant Power (FRAP) Assay

The FRAP activity was measured based on a previous study [29]. In brief, 0.05 mL of FeCl_3_ solution (4 mM in 0.05 M HCl) was added to an equal portion of the sample (prepared as described in Section 2.4.7) and the solution was incubated for 30 min at 37 °C. Then, 0.90 mL of TPTZ solution (1 mM in 0.05 M HCl) was added, and after 5 min, the absorbance at 620 nm was recorded. A calibration curve was prepared using ascorbic acid as the standard compound. Results (*P*_R_) were expressed as μmoL of ascorbic acid equivalents (AAE) per g dw, using the following equation:

(3) $$P_{R}\;(\mu \mathrm{moL}\;\mathrm{AAE}/g\;\mathrm{dw})=\frac{C_{\mathrm{AA}}\;\times \;V}{w}$$

where *V* is the volume of the extraction medium (in L) and *w* is the dry weight of the sample (in g).

### 2.5. Statistical Analysis

For the survival and growth study of the larvae, a total of 8 measurements (four replicate trays and 2 subsamples per tray) were taken. For the proximate composition analyses, all determinations were carried out in triplicates, using three samples in each, resulting in a total of nine measurements. Results were expressed as mean values of the nine measurements ± standard error of mean (SEM). Next, the Kolmogorov–Smirnov test was carried out to examine whether the results were normally distributed. Statistically significant differences (*p* < 0.05) between the samples were assessed with the Kruskal–Wallis test. The statistical analyses were performed using SPSS (version 29) (SPSS Inc., Chicago, IL, USA) software.

## 3. Results and Discussion

### 3.1. Survival and Growth of T. molitor Larvae

The first parameter to consider when examining a potential feed for insect larvae is whether it has an impact on their survival. To this end, the effect of the addition of different orange albedo powder proportions in wheat bran was examined, in terms of larval survival. Wheat bran was selected as the control feed since it is a commonly used substrate that promotes rapid growth and ensures high survival and high larval weight gain [30]. Larval survival was evaluated every week, starting from the end of the third week, until the end of the sixth week (before larvae pupate). The % survival rates of the larvae at each evaluation interval are given in Table 1. As can be seen, no significant differences (*p* > 0.05) were recorded among the control samples within the 4 weeks. This was also the case with all the examined additions of the orange albedo powder. Therefore, it can be concluded that the use of orange albedo powder is safe for the production of *T. molitor* larvae. This is an encouraging finding, since in a previous study, the use of the essential oils from orange peels was found to reduce the survival of *T. molitor* adults by 12.4% [31]. However, this was not proven to be a hindrance in our case, for two main reasons. First, the essential oil of the orange peel is located mainly in the outer part (i.e., flavedo), where the oil glands are located [32]. Since in our case, the albedo part was used (since the flavedo part is used as zest for culinary reasons), the presence of essential oils is minimal. Secondly, in order to produce the orange albedo powder, the albedos were subjected to freeze-drying, which is known to reduce the amount of essential oils in plant tissues [33]. Additionally, it is noteworthy that in all cases, the larvae exhibited a preference toward albedo powder, compared to the wheat bran, suggesting that the larvae, through self-selection, can better regulate their nutritional needs [10].

**Figure 1:**
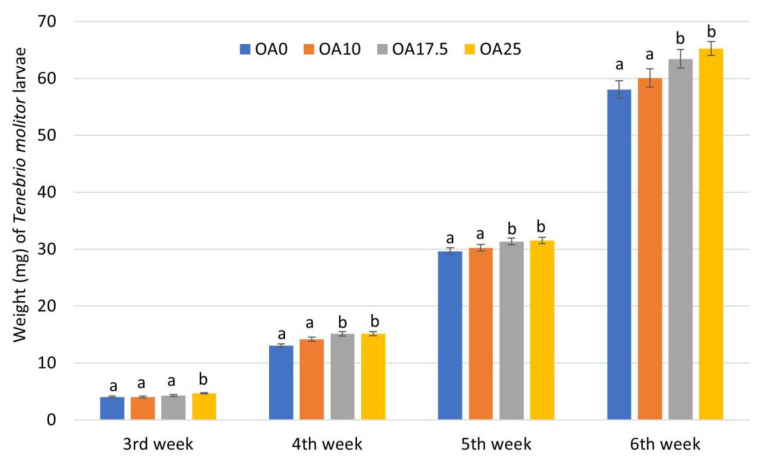
Individual weight (mg) of *Tenebrio molitor* larvae fed for six weeks with wheat bran (control) (OA0) and bran fortified with different rates of albedo orange peel waste (10 (OA10), 17.5 (OA17.5), and 25% (OA25)) (n = 8). Statistically significant differences (*p* < 0.05) are denoted with different superscript letters.

The next crucial parameter is to examine the effect of orange albedo powder on the growth of the *T. molitor* larvae. The individual larval weight for each dietary treatment is provided in Figure 1. It is noteworthy that the weight difference of the larvae upon birth was very low (<0.00010); therefore, all differences in the weight were attributed to the different treatments. The individual larval weight at the initiation of the trial was 1.07 mg. Based on the results, not only did the orange albedo not hinder larval growth, but in most cases, it promoted their growth. More specifically, after 1 week of consuming the fortified feeds (third week), a significant (*p* < 0.05) weight increase was recorded for the larvae fed with wheat bran supplemented with 25% orange albedo. In the two other feeds (10% and 17.5% albedo), no significant increase was recorded compared to the control. By week four, the larvae that consumed the feed that contained 17.5% and 25% albedo were found to be significantly (*p* < 0.05) increased, when compared to the control, as an increase in the larval weight of up to 14.7% was recorded. The same trend was also observed at the next two evaluation intervals (weeks five and six). The larvae fed with the two abovementioned diets were found to be significantly (*p* < 0.05) larger, compared to the control larvae.

To date, quite a few by-products have been examined as feed for mealworm larvae [15]. For instance, feeding biscuits as a single substrate or supplementing them with used cereals or bread resulted in reduced larval growth and development [17]. On the contrary, chicken feed, rapeseed meal, wheat bran, and sunflower with willow leaves, were found to promote growth, with bran being the optimum [34]. As such, the increased growth of larvae upon any feed, supplemented with by-products, cannot be taken for granted. According to our results, the orange albedo can be used as an alternative option to supplement feed, since it does not reduce larval survival and it promotes their growth above a concentration of 17.5% *w*/*w*.

### 3.2. Evaluation of the Nutritional Value of the Larvae

Before examining the proximate composition of the larvae, the water content of the samples was evaluated. In all cases, it was found that the samples contained water ranging between 37 and 38%. 

#### 3.2.1. Proximate Composition

The main reason why insects have attracted the interest of researchers and have started to be exploited in Western countries as edible food is that they have a high protein content [35]. In principle, *T. molitor* larvae are known to have a high protein content. For instance, Costa et al. [36] estimated the protein content of *T. molitor* larvae to be 13.7 g per 100 g of their net weight, Nowak et al. [37] reported values between 13.68 and 22.32 g/100 g, and finally, Ghaly et al. [38] reported values between 24.3 and 27.6 g per 100 g of their net weight. Therefore, in the present study, the larvae fed with different diets were examined for their crude protein content. The proximate composition of the *T. molitor* larvae fed the different dietary treatments by the end of the sixth week are shown in Table 2. Our results illustrate that the increase in the percentage of the albedo in the feed caused a rather proportional increase in the protein content of the larvae. More specifically, the larvae fed with wheat bran containing 10% orange albedo contained 13.2% more proteins (*p* < 0.05) compared to the control. Likewise, a statistically significant increase (*p* < 0.05) of 24.6% and 32% was registered when the larvae were fed with 17.5% and 25% orange albedo added to the wheat bran. This is a considerable increase in the protein content, given that the orange albedo proved to be a very poor source of crude protein (~1.5%), compared to previous reports [39]. For instance, Mancini et al. [17] found that feeding *T. molitor* larvae with bread remains or soon-to-expire cookies resulted in a decrease in the protein content of the larvae, despite the ~10% protein content of the feed. In our case, orange albedo promoted larval growth and protein formation. Although this may seem contradictory, the higher protein content of the orange albedo-fed larvae may be due to the increased feed assimilability and feed-protein conversion rates and not due to the increased protein content of the feed [40]. Therefore, orange albedo proved to be an efficient means to increase the protein content of the larvae. Thus, the orange albedo-fed larvae can be considered more beneficial for human consumption, due to the increased protein content.

Next, the content of the mealworm larvae in carbohydrates was evaluated, despite their relatively low content that often results in overlooking this parameter [21]. As can be seen in Table 2, the increase in the content of orange albedo caused a decrease in carbohydrates. In this context, a decrease in the *T. molitor* larvae carbohydrate content of 15.4, 30.1, and 31.2% (significant for *p* < 0.05) was recorded for OA10, OA17.5, and OA25, respectively. This decrease in the carbohydrate content may be associated with the increased growth rate. Since the larvae are preparing gradually for metamorphosis, proteins and lipids are more important and therefore, more carbohydrates are needed to meet the energy demands for growth [10]. The relatively low carbohydrate content of the larvae may be beneficial for the human diet, since carbohydrates can easily be consumed by other sources and therefore, the low content assists in an easier diet balance. Moreover, out of the carbohydrates contained in the larvae, about 3% is due to soluble sugars, while a considerable amount comprises chitin, which serves as an excellent source of fiber [21]. 

Additionally, the crude ash content of the samples was determined (Table 2). The control larvae were found to contain 1.06% crude ash. As the content of the feed in orange albedo increased, an increase in the crude ash of the larvae was also recorded. More specifically, a statistically significant (*p* < 0.05) increase of 10, 21.6, and 26.5% was recorded in the crude ash content for OA10, OA17.5, and OA25, respectively. The above results corroborate that the larvae fed with the orange albedo are a better source of minerals, compared to the control larvae. The crude ash content of the samples was also found to be comparable to that of previous studies. For instance, Siemianowska et al. [41] reported an ash content of 1.55%, Costa et al. [36] reported a content of 1.23%, while in other studies, the content ranged between 0.9 and 3.81% [37], or between 1.8 and 2.2% [38]. The increased content of ash in the larvae can assist human nutrition [38]. 

Fat is another key nutrient that contributes to proper human nutrition [42]. In our results, the control larvae contained 42.16% crude fat (Table 2). When orange albedo was added to the feed at percentages of 10% and 17.5%, no significant (*p* > 0.05) difference was recorded when compared to the control sample. However, when orange albedo was added at 25%, the larvae were found to contain 3.32% less fat, as compared with the respective figures from the control larvae. This decrease in the fat content may be attributed to the low fat content of the orange albedo (~1.47% [43]). In previous studies, various percentages have been reported for the fat content of the larvae, ranging from 32.7% [44,45] up to 41% [46]. Therefore, the larvae in our case have comparable fat content, when compared to previous studies. 

In all cases, the main fatty acids were found to be oleic acid (C18:1), ranging between 39.07% and 43.68%, followed by linoleic acid (C18:2), ranging between 27.34% and 29.68%, and finally, palmitic acid (C16:0), ranging between 21.39% and 23.22% (Table 3). Oleic acid has many beneficial properties for human health, such as the prevention–suppression of breast cancer and rheumatoid arthritis [47]. The *T. molitor* larvae were also rich in linoleic acid [36], which is one of the two essential fatty acids that the human body cannot synthesize from other food components [48]. Animal sources that are considered rich in omega-6 fatty acids are chicken and egg yolk. In particular, chicken fat may contain 18–23% and egg yolk 16% linoleic acid [49], whereas *T. molitor* larvae were found to contain nearly two times more linoleic acid [36]. It is noteworthy that the amount of saturated fatty acids (SFAs) was found to decrease in the presence of orange albedo in the feed. More specifically, in all cases, a statistically significant decrease (*p* < 0.05) of ~16% was recorded. As regards the monounsaturated fatty acids (MUFAs), although an increase of 2–3% was recorded in the larvae fed with orange albedo, this increase was not found to be significant (*p* > 0.05). However, a significant increase (*p* < 0.05) of up to 8.6% in the content of polyunsaturated fatty acids (PUFAs) was recorded in all cases of the larvae fed with orange albedo. Considering the above, the PUFA: SFA ratio was calculated for all samples to assess their impact on cardiovascular health [50]. The control larvae were found to have a ratio of 0.87, whereas for the orange albedo-fed larvae, in all cases, the ratio was above 1. Due to this, their consumption is likely to have a more positive effect on the cardiovascular system. In comparison, the pig, lamb, and cattle meat correspond to a ratio between 0.13 and 0.48, while fish, a well-renounced source of fatty acids, has a PUFA: SFA ratio of 0.8–1.6 [50], suggesting that the larvae are a comparable source of fatty acids with potential beneficial effects. The ccalculated oxidizability value (COX) was also measured for all samples. As can be seen from the results, a significant increase (*p* < 0.05) in the COX value was recorded in all cases of the larvae fed with orange albedo. Therefore, the oil extracted from the larvae exhibits better oxidative stability, and thus, has a longer shelf life.

#### 3.2.2. Content of *T. molitor* Larvae in Vitamins A and C

Vitamin A is a group of fat-soluble retinoids, including retinol and retinyl esters [51]. Vitamin A can be found either preformed or as a provitamin A, known as carotenoids. The latter are plant pigments that are convertible to vitamin A in the gut [52]. Vitamin A is well-known for its anti-inflammatory activity, whereas it also assists the functions of the immune system by supporting the growth and distribution of T cells, helps the preservation of epithelial and mucosal tissues, and promotes fetal development and the well-being of the reproductive system [53,54,55,56]. Given that fat-soluble vitamins, such as vitamin A, are detected in low amounts in edible insects, such as *T. molitor*, increasing their amounts would significantly enhance their nutritional value. In general, many insect species have been found to contain low amounts of vitamin A, so various attempts have been made to increase it [57]. For instance, Oonincx et al. [58] tried to increase the vitamin A content of the migratory locust, *Locusta migratoria* (L.) (Orthoptera: Acrididae), by enriching its diet with fresh carrots. The results showed that insects that consumed carrots had higher levels of vitamin A than those that consumed their standard diet; however, the increase was not as high as expected. A similar piece of research was also carried out on *T. molitor* larvae using commercial supplements; however, the larvae were found to contain marginally detectable vitamin A amounts [59]. 

Orange albedo is a good source of β-carotene and vitamin A. According to the results of our previous study, the orange albedo was found to contain ~36 μg/g dw of β-carotene [28]. Therefore, it was expected that the larvae would contain an increased amount of β-carotene and vitamin A after being fed with orange albedos. The respective results can be seen in Table 4. It is obvious that in both cases, as the amount of orange albedo in the feed increased, an increase in the amounts of β-carotene and vitamin A was also recorded. More specifically, when the larvae were fed a diet containing 10, 17.5, or 25% orange albedo, a significant (*p* < 0.05) increase of 85, 144, and 198%, respectively, was recorded in the β-carotene and vitamin A content. Therefore, orange albedos resulted in a significant increase in the β-carotene and vitamin A content of the larvae. 

As regards the content of the larvae in vitamin C, an increase in the amount of vitamin C detected in the larvae was recorded, upon feeding with orange albedo (Table 4). When the larvae were fed a diet containing 10% orange albedo, a significant (*p* < 0.05) increase of 25% was recorded in the vitamin C content. In the case of 17.5% orange albedo, an increase of 40% (significant for *p* < 0.05) was recorded, whereas in the case of 25% albedo, an increase of 46% (significant for *p* < 0.05) was also recorded. Therefore, feeding with orange albedo also proved beneficial in this case.

#### 3.2.3. Antioxidant Activity of the Larvae Extracts

Orange albedo contains many polyphenolic compounds that exhibit bioactivity [28]. As such, it was expected that when the larvae were fed with orange albedos, their content in polyphenols would increase. This hypothesis was validated by our results. It was found that when the larvae were fed with a diet containing 0 (control), 10, 17.5, and 25% orange albedo, the polyphenol content was found to be 59.67 ± 0.43, 69.01 ± 0.58, 73.64 ± 1.60, and 78.67 ± 1.07 mg/g polyphenols, corresponding to a significant (*p* < 0.05) increase of 15.6, 23.4, and 31.8% when compared to the control, respectively. Therefore, orange albedo significantly increased the polyphenol content of the larvae, bestowing them with potential benefits for human consumption. The reported values for the TPC were similar to that of previous reports [60,61]. Finally, the FRAP values of the larvae were measured. It was found that the control larvae exhibited a FRAP value of 119.39 ± 1.97 μmoL AAE/g. For the OA10 larvae, the FRAP value was found to be 140.01 ± 6.57 μmoL AAE/g (a statistically significant, *p* < 0.05, increase of 17.3%). In the case of OA17.5, the FRAP value was found to be 143.64 ± 2.22 μmoL AAE/g (a statistically significant, *p* < 0.05, increase of 20.3%), while in the case of OA25, the FRAP value was found to be 145.97 ± 2.68 μmoL AAE/g (a statistically significant, *p* < 0.05, increase of 22.2%). The enhanced content in polyphenols resulted in increased antioxidant activity, which is beneficial for human consumption, and it may prove beneficial for the protection of the nutrients contained in the larvae from oxidation. The FRAP value for the control larvae (OA0) was found to be nearly half when compared to that reported by Sánchez-Muros et al. [62]. However, this difference may be attributed to the different diets of the larvae (cereal brans were used in the previous study, compared to wheat bran only in our case). 

## 4. Conclusions

Supplementation of the standard diet of *T. molitor* larvae (wheat bran) with orange albedo was found to be highly beneficial for larval growth and performance, as well as for their nutritional value. Without adversely affecting larval survival, the addition of 17.5% and 25% orange albedo in the feed resulted in a noticeable increase in larval growth. Moreover, the nutritional value of the larvae was significantly enhanced. Even with the smallest addition of orange albedo, a major increase in the crude protein content was recorded, accompanied by an increase in the ash content. On the contrary, the content of fat and carbohydrates decreased as the amount of orange albedo increased. In addition, a further enhancement of the nutritional value was recorded, as the amount of SFAs decreased and the amount of MUFAs and PUFAs increased. Moreover, increased contents of vitamins A and C, as well as polyphenols and antioxidant activity, were recorded. Overall, the use of 25% waste orange peel albedos is highly recommended for the rearing of *T. molitor* larvae, since it combines many advantages that simultaneously correspond to various quantitative and qualitative parameters, such as larval growth and larval composition. Apart from this, the addition of orange peel albedos to the diet of *T. molitor* will further reduce the cost of mass-rearing protocols, rendering the entire production procedure a more economically preferable option for insect rearing. Additionally, by feeding larvae with orange albedos, the circular economy is further promoted, since albedos constitute a by-product that is rarely reused, which constitutes an additional advantage in terms of improving the environmental compatibility indicators in insect farming.

## Figures and Tables

**Table 1:** Survival (%) of *Tenebrio molitor* larvae (±SEM) fed for six weeks with wheat bran (control) (OA0) and bran fortified with different rates of albedo orange peel waste (10 (OA10), 17.5 (OA17.5), and 25% (OA25)) (n = 8).

Diets	Survival (%)			
	Third Week	Fourth Week	Fifth Week	Sixth Week
OA0	86.64 ± 2.15	86.36 ± 2.96	82.24 ± 2.50	81.16 ± 2.79
OA10	84.48 ± 2.02	81.44 ± 2.56	80.80 ± 2.94	79.88 ± 2.14
OA17.5	85.16 ± 2.23	83.84 ± 2.21	82.44 ± 2.76	82.16 ± 2.21
OA25	94.32 ± 3.68	90.84 ± 4.21	88.60 ± 2.14	88.04 ± 2.98

**Table 2:** Proximate composition of *Tenebrio molitor* larvae fed for six weeks with wheat bran (control) (OA0) and bran fortified with different rates of albedo orange peel waste (10 (OA10), 17.5 (OA17.5), and 25% (OA25)) (n = 9).

% Composition of Dry Weight	OA0	OA10	OA17.5	OA25
Crude protein	32.25 ± 0.94 ^a,^*	36.5 ± 1.28 ^b^	40.18 ± 1.65 ^c^	44.2 ± 2.08 ^d^
Crude fat	42.16 ± 1.12 ^a^	41.97 ± 1.98 ^a^	41.12 ± 1.03 ^a^	38.84 ± 0.81 ^b^
Carbohydrates	22.8 ± 0.89 ^a^	19.28 ± 0.75 ^b^	15.91 ± 0.46 ^c^	15.67 ± 0.56 ^c^
Crude ash	1.06 ± 0.03 ^a^	1.17 ± 0.03 ^b^	1.29 ± 0.05 ^c^	1.34 ± 0.06 ^c^
Energy (kcal/100 g)	599.64 ± 23.07 ^a^	600.85 ± 22.58 ^a^	594.44 ± 24.45 ^a^	590.24 ± 15.04 ^a^

* Within each line, statistically significant differences (*p* < 0.05) are denoted with different superscript letters.

**Table 3:** Fatty acid composition of *Tenebrio molitor* larvae fed for six weeks with wheat bran (control) (OA0) and bran fortified with different rates of albedo orange peel waste (10 (OA10), 17.5 (OA17.5), and 25% (OA25)) (n = 9).

Fatty Acid (%)	Diets			
	OA0	OA10	OA17.5	OA25
C12:0	0.16 ± 0.01 ^b,c,^*	0.15 ± 0.01 ^c^	0.19 ± 0.01 ^a^	0.17 ± 0.01 ^b^
C14:0	2.32 ± 0.11 ^b^	2.71 ± 0.08 ^a^	2.68 ± 0.06 ^a^	2.70 ± 0.06 ^a^
C16:0	21.39 ± 0.98 ^a^	23.27 ± 1.16 ^a^	23.03 ± 0.71 ^a^	23.22 ± 1.46 ^a^
C16:1	0.12 ± 0.01 ^a,b^	0.10 ± 0.01 ^c^	0.11 ± 0.01 ^b,c^	0.12 ± 0.01 ^a^
C18:0	0.13 ± 0.01 ^d^	0.17 ± 0.01 ^c^	0.19 ± 0.01 ^b^	0.20 ± 0.01 ^a^
C18:1	39.07 ± 2.89 ^b^	44.1 ± 2.82 ^a^	43.79 ± 0.18 ^a^	43.68 ± 2.10 ^a^
C18:2 (ω-6)	27.34 ± 0.57 ^b^	29.28 ± 0.73 ^a^	29.84 ± 1.1 ^a^	29.68 ± 0.65 ^a^
C20:0	1.36 ± 0.05	nd **	nd	nd
C18:3 (ω-3)	0.08 ± 0.01 ^b^	0.11 ± 0.01 ^a^	0.09 ± 0.01 ^b^	0.11 ± 0.01 ^a^
C22:0	6.04 ± 0.31 ^a^	0.03 ± 0.01 ^b^	0.02 ± 0.01 ^b^	0.03 ± 0.01 ^b^
C22:1	1.82 ± 0.13 ^a^	0.09 ± 0.01 ^b^	0.07 ± 0.01 ^b^	0.08 ± 0.01 ^b^
C24:0	0.16 ± 0.01	nd	nd	nd
*∑* SFA ^1^	31.57 ± 1.48 ^a^	26.32 ± 1.26 ^b^	26.11 ± 0.78 ^b^	26.32 ± 1.54 ^b^
*∑* MUFA ^2^	41.01 ± 3.02 ^a^	44.29 ± 2.83 ^a^	43.97 ± 0.18 ^a^	43.88 ± 2.11 ^a^
*∑* PUFA ^3^	27.42 ± 0.58 ^b^	29.39 ± 0.74 ^a^	29.93 ± 1.11 ^a^	29.79 ± 0.66 ^a^
PUFA: SFA ratio	0.87 ± 0.02 ^b^	1.12 ± 0.03 ^a^	1.15 ± 0.01 ^a^	1.13 ± 0.04 ^a^
MUFA: PUFA ratio	1.49 ± 0.08 ^a^	1.51 ± 0.06 ^a^	1.47 ± 0.05 ^a^	1.47 ± 0.04 ^a^
ω-6: ω-3 ratio	331.29 ± 6.96 ^a^	261.62 ± 8.92 ^b^	344.68 ± 9.69 ^a^	277.82 ± 3.62 ^b^
COX ^4^	3.22 ± 0.09 ^b^	3.48 ± 0.11 ^a^	3.53 ± 0.12 ^a^	3.52 ± 0.09 ^a^

* Within each line, statistically significant differences (*p* < 0.05) are denoted with different superscript letters. ** nd: not detected. ^1^ SFAs, saturated fatty acids (%): SUM of C12:0, lauric acid; C14:0, myristic acid; C16:0, palmitic acid; C18:0, stearic acid; C20:0, arachidic acid; C22:0, behenic acid; C24:0, lignoceric acid. ^2^ MUFAs, monounsaturated fatty acids (%): SUM of C16:1, palmitoleic acid; C18:1, oleic acid; C22:1, erucic acid. ^3^ PUFAs, polyunsaturated fatty acids (%): SUM of C18:2, ω-6, linoleic acid; C18:3, ω-3, linolenic acid. ^4^ COX, calculated oxidizability value.

**Table 4:** Content of *Tenebrio molitor* larvae fed for six weeks with wheat bran (control) (OA0) and bran fortified with different rates of albedo orange peels waste (10 (OA10), 17.5 (OA17.5), and 25% (OA25)) in β-carotene, vitamin A, and vitamin C (n = 9).

Diets	β-Carotene (µg/g)	Vitamin A (μg RAE/100 g)	Vitamin C (μg/g)
OA0	3.83 ± 0.06 ^a,^*	11.41 ± 0.19 ^a^	200.37 ± 6.44 ^a^
OA10	7.10 ± 0.29 ^b^	21.17 ± 0.86 ^b^	251.44 ± 4.63 ^b^
OA17.5	9.45 ± 0.01 ^c^	28.19 ± 0.04 ^c^	282.50 ± 2.88 ^c^
OA25	11.43 ± 0.1 ^d^	34.08 ± 0.29 ^d^	292.90 ± 1.42 ^d^

* Within each column, statistically significant differences (*p* < 0.05) are denoted with different superscript letters.

## Data Availability

All the data are contained within the article.

## References

[1] Boland M.J., Rae A.N., Vereijken J.M., Meuwissen M.P.M., Fischer A.R.H., van Boekel M.A.J.S., Rutherfurd S.M., Gruppen H., Moughan P.J., Hendriks W.H. (2013). The future supply of animal-derived protein for human consumption. Trends Food Sci. Technol..

[2] Foley J.A., Ramankutty N., Brauman K.A., Cassidy E.S., Gerber J.S., Johnston M., Mueller N.D., O’Connell C., Ray D.K., West P.C. (2011). Solutions for a cultivated planet. Nature.

[3] Grasty S., FAO (1999). Reducing Enteric Methane and Livelihoods Win-Win Opportunities for Farmers.

[4] Patel S., Suleria H.A.R., Rauf A. (2019). Edible insects as innovative foods: Nutritional and functional assessments. Trends Food Sci. Technol..

[5] Premalatha M., Abbasi T., Abbasi T., Abbasi S.A. (2011). Energy-efficient food production to reduce global warming and ecodegradation: The use of edible insects. Renew. Sustain. Energy Rev..

[6] Errico S., Spagnoletta A., Verardi A., Moliterni S., Dimatteo S., Sangiorgio P. (2022). *Tenebrio molitor* as a source of interesting natural compounds, their recovery processes, biological effects, and safety aspects. Compr. Rev. Food Sci. Food Saf..

[7] European Union Commission (2021). Commission Implementing Regulation (EU) 2021/882 Authorising the Placing on the Market of Dried Tenebrio molitor Larva as a Novel Food under Regulation (EU) 2015/2283 of the European Parliament and of the Council, and Amending Commission Implementing Regulation (EU) 2017/2470 (Text with EEA Relevance).

[8] Melo V., Garcia M., Sandoval H., Jiménez H.D., Calvo C. (2011). Quality proteins from edible indigenous insect food of latin America and Asia. Emirates J. Food Agric..

[9] van Huis A., Oonincx D.G.A.B. (2017). The environmental sustainability of insects as food and feed. A review. Agron. Sustain. Dev..

[10] Kröncke N., Benning R. (2022). Self-Selection of Feeding Substrates by *Tenebrio molitor* Larvae of Different Ages to Determine Optimal Macronutrient Intake and the Influence on Larval Growth and Protein Content. Insects.

[11] Oonincx D.G.A.B., Finke M.D. (2021). Nutritional value of insects and ways to manipulate their composition. J. Insects Food Feed..

[12] Punzo F., Mutchmor J.A. (1980). Effects of Temperature, Relative Humidity and Period of Exposure on the Survival Capacity of *Tenebrio molitor* (Coleoptera: Tenebrionidae). Survival.

[13] Cotton R.T., Ashby W. (1952). Insect pests of stored grains and seed. Insects: The Yearbook of Agriculture.

[14] Rumbos C.I., Karapanagiotidis I.T., Mente E., Psofakis P., Athanassiou C.G. (2020). Evaluation of various commodities for the development of the yellow mealworm, *Tenebrio molitor*. Sci. Rep..

[15] Van Peer M., Frooninckx L., Coudron C., Berrens S., Álvarez C., Deruytter D., Verheyen G., Van Miert S. (2021). Valorisation potential of using organic side streams as feed for *Tenebrio molitor*, *Acheta domesticus* and *Locusta migratoria*. Insects.

[16] Pfaltzgraff L.A., De Bruyn M., Cooper E.C., Budarin V., Clark J.H. (2013). Food waste biomass: A resource for high-value chemicals. Green Chem..

[17] Mancini S., Fratini F., Turchi B., Mattioli S., Dal Bosco A., Tuccinardi T., Nozic S., Paci G. (2019). Former foodstuff products in *Tenebrio molitor* rearing: Effects on growth, chemical composition, microbiological load, and antioxidant status. Animals.

[18] Morales-Ramos J.A., Rojas M.G., Kelstrup H.C., Emery V. (2020). Self-selection of agricultural by-products and food ingredients by *Tenebrio* molitor (Coleoptera: Tenebrionidae) and impact on food utilization and nutrient intake. Insects.

[19] Rumbos C.I., Bliamplias D., Gourgouta M., Michail V., Athanassiou C.G. (2021). Rearing *Tenebrio molitor* and *Alphitobius diaperinus* Larvae on Seed Cleaning Process Byproducts. Insects.

[20] Bradford M.M. (1976). A rapid and sensitive method for the quantitation of microgram quantities of protein utilizing the principle of protein-dye binding. Anal. Biochem..

[21] Son Y.J., Hwang I.K., Nho C.W., Kim S.M., Kim S.H. (2021). Determination of carbohydrate composition in mealworm (*Tenebrio molitor* L.) larvae and characterization of mealworm chitin and chitosan. Foods.

[22] European Union Commission (2002). Commission Regulation (EC) No 796/2002 of 6 May 2002 amending Regulation (EEC) No 2568/91 on the Characteristics of Olive Oil and Olive-Pomace Oil and on the Relevant Methods of Analysis and the Additional Notes in the Annex to Council Regulation (EEC) No 2658/87 on the Tariff and Statistical Nomenclature and on the Common Customs Tariff.

[23] Lalas S., Gortzi O., Athanasiadis V., Dourtoglou E., Dourtoglou V. (2012). Full Characterisation of Crambe abyssinica Hochst. Seed Oil. J. Am. Oil Chem. Soc..

[24] Ourailoglou D., Athanasiadis V., Bozinou E., Salakidou C., Evmorfopoulos E., Lalas S. (2021). Manufacturing Process and Physicochemical Analysis of Kariki: A Traditional Cheese from The Island of Tinos, Greece. Int. Food Res. J..

[25] Fatemi S.H., Hammond E.G. (1980). Analysis of oleate, linoleate and linolenate hydroperoxides in oxidized ester mixtures. Lipids.

[26] Ocampo E.T.M., Libron J.A.M.A., Guevarra M.L.D., Mateo J.M.C. (2020). Phytochemical screening, phenolic acid profiling and antioxidant activity analysis of peels from selected mango (*Mangifera* spp.) genotypes in the Philippines. Food Res..

[27] Jagota S.K., Dani H.M. (1982). A new colorimetric technique for the estimation of vitamin C using Folin phenol reagent. Anal. Biochem..

[28] Athanasiadis V., Chatzimitakos T., Kotsou K., Palaiogiannis D., Bozinou E., Lalas S.I. (2022). Optimization of the Extraction Parameters for the Isolation of Bioactive Compounds from Orange Peel Waste. Sustainability.

[29] Athanasiadis V., Palaiogiannis D., Poulianiti K., Bozinou E., Lalas S.I., Makris D.P. (2022). Extraction of Polyphenolic Antioxidants from Red Grape Pomace and Olive Leaves: Process Optimization Using a Tailor-Made Tertiary Deep Eutectic Solvent. Sustainability.

[30] LeCato G., Flaherty B. (1974). Description of eggs of selected species of stored-product insects (Coleoptera and Lepidoptera). J. Kansas Entomol. Soc..

[31] Abdelatti Z.A.S., Hartbauer M. (2020). Plant oil mixtures as a novel botanical pesticide to control gregarious locusts. J. Pest Sci. (2004).

[32] Yang Y., Wang X., Zhao C., Tian G., Zhang H., Xiao H., He L., Zheng J. (2017). Chemical Mapping of Essential Oils, Flavonoids and Carotenoids in Citrus Peels by Raman Microscopy. J. Food Sci..

[33] Hazarika U., Gosztola B. (2020). Lyophilization and its Effects on the Essential Oil Content and Composition of Herbs and Spices—A Review. Acta Sci. Pol. Technol. Aliment..

[34] Bordiean A., Krzyżaniak M., Stolarski M.J. (2022). Bioconversion Potential of Agro-Industrial Byproducts by *Tenebrio molitor*—Long-Term Results. Insects.

[35] Rumpold B.A., Schlüter O.K. (2013). Nutritional composition and safety aspects of edible insects. Mol. Nutr. Food Res..

[36] Costa S., Pedro S., Lourenço H., Batista I., Teixeira B., Bandarra N.M., Murta D., Nunes R., Pires C. (2020). Evaluation of Tenebrio molitor larvae as an alternative food source. NFS J..

[37] Nowak V., Persijn D., Rittenschober D., Charrondiere U.R. (2016). Review of food composition data for edible insects. Food Chem..

[38] Ghaly A.E., Alkoaik F.N. (2009). The yellow mealworm as a novel source of protein. Am. J. Agric. Biol. Sci..

[39] Ben-Shalom N., Pinto R., Berman M. (1985). Polysaccharides and proteins in the orange albedo and in the aqueous extract of the tissue. Can. Inst. Food Sci. Technol. J..

[40] Yu X., He Q., Wang D. (2021). Dynamic Analysis of Major Components in the Different Developmental Stages of *Tenebrio molitor*. Front. Nutr..

[41] Siemianowska E., Kosewska A., Aljewicz M., Skibniewska K.A., Polak-Juszczak L., Jarocki A., Jędras M. (2013). Larvae of mealworm (*Tenebrio molitor* L.) as European novel food. Agric. Sci..

[42] Kaur N., Chugh V., Gupta A.K. (2014). Essential fatty acids as functional components of foods- a review. J. Food Sci. Technol..

[43] Baioumy A.A., Abedelmaksoud T.G. (2021). Quality properties and storage stability of beef burger as influenced by addition of orange peels (albedo). Theory Pract. Meat Process..

[44] Ravzanaadii N., Kim S.-H., Choi W.-H., Hong S.-J., Kim N.-J. (2012). Nutritional Value of Mealworm, *Tenebrio molitor* as Food Source. Int. J. Ind. Entomol..

[45] Ghosh S., Lee S.M., Jung C., Meyer-Rochow V.B. (2017). Nutritional composition of five commercial edible insects in South Korea. J. Asia. Pac. Entomol..

[46] Caparros Megido R., Poelaert C., Ernens M., Liotta M., Blecker C., Danthine S., Tyteca E., Haubruge É., Alabi T., Bindelle J. (2018). Effect of household cooking techniques on the microbiological load and the nutritional quality of mealworms (*Tenebrio molitor* L. 1758). Food Res. Int..

[47] Pastor R., Bouzas C., Tur J.A. (2021). Beneficial effects of dietary supplementation with olive oil, oleic acid, or hydroxytyrosol in metabolic syndrome: Systematic review and meta-analysis. Free Radic. Biol. Med..

[48] Burr M., Miller S. (1932). Fatty Acids in Nutrition. J. Biol.

[49] Nutter M.K., Lockhart E.E., Harris R.S. (1943). The chemical composition of depot fats in chickens and turkeys. Oil Soap.

[50] Chen J., Liu H. (2020). Nutritional indices for assessing fatty acids: A mini-review. Int. J. Mol. Sci..

[51] Huth E.J. (1988). A Library for Internists VI Recommended by the American College of Physicians. Ann. Intern. Med..

[52] Solomons N.W., Orozco M. (2003). Alleviation of vitamin A deficiency with palm fruit and its products. Asia Pac. J. Clin. Nutr..

[53] Sommer A. (2008). Vitamin A deficiency and clinical disease: An historical overview. J. Nutr..

[54] Liang Y., Yi P., Wang X., Zhang B., Jie Z., Soong L., Sun J. (2020). Retinoic Acid Modulates Hyperactive T Cell Responses and Protects Vitamin A–Deficient Mice against Persistent Lymphocytic Choriomeningitis Virus Infection. J. Immunol..

[55] Ross S.A., McCaffery P.J., Drager U.C., De Luca L.M. (2000). Retinoids in embryonal development. Physiol. Rev..

[56] Bastos Maia S., Rolland Souza A.S., Costa Caminha M.d.F., Lins da Silva S., Callou Cruz R.d.S.B.L., Carvalho dos Santos C., Batista Filho M. (2019). Vitamin A and Pregnancy: A Narrative Review. Nutrients.

[57] Finke M.D. (2002). Complete nutrient composition of commercially raised invertebrates used as food for insectivores. Zoo Biol..

[58] Oonincx D.G.A.B., Van Der Poel A.F.B. (2011). Effects of diet on the chemical composition of migratory locusts (*Locusta migratoria*). Zoo Biol..

[59] Finke M.D. (2015). Complete nutrient content of four species of commercially available feeder insects fed enhanced diets during growth. Zoo Biol..

[60] Antonopoulou E., Panteli N., Feidantsis K., Mastoraki M., Koutsogeorgiou E., Grivaki E., Papagrigoriou T., Christias S., Chatzifotis S., Lazari D. (2022). Carob (*Ceratonia siliqua*) as Functional Feed Is Beneficial in Yellow Mealworm (*Tenebrio molitor*) Rearing: Evidence from Growth, Antioxidant Status and Cellular Responses. Antioxidants.

[61] Keil C., Grebenteuch S., Kröncke N., Kulow F., Pfeif S., Kanzler C., Rohn S., Boeck G., Benning R., Haase H. (2022). Systematic Studies on the Antioxidant Capacity and Volatile Compound Profile of Yellow Mealworm Larvae (*Tenebrio molitor* L.) under Different Drying Regimes. Insects.

[62] Sánchez-Muros M.J., de Haro C., Sanz A., Trenzado C.E., Villareces S., Barroso F.G. (2016). Nutritional evaluation of *Tenebrio molitor* meal as fishmeal substitute for tilapia (*Oreochromis niloticus*) diet. Aquac. Nutr..

